# Case Series of Quadricuspid Aortic Valve

**DOI:** 10.7759/cureus.28888

**Published:** 2022-09-07

**Authors:** Ihtisham Khalid, Hasnan M Ijaz, Poonam Choudhry, Aaiz Hussain, James McAlister III, Ahmed Mahmood, Mustafa Rahim, Henry Cusnir

**Affiliations:** 1 Internal Medicine, Hospital Corporation of America (HCA) Florida Westside Hospital, Plantation, USA; 2 Dr. Kiran C. Patel College of Allopathic Medicine, Nova Southeastern University, Davie, USA; 3 Cardiology, Corpus Christi Medical Center, Corpus Christi, USA; 4 Internal Medicine, Raleigh General Hospital, Beckley, USA; 5 Cardiology, Hospital Corporation of America (HCA) Florida Westside Hospital, Plantation, USA

**Keywords:** echocardiography, aortic valve repair, aortic regurgitation, aortic vale, quadricuspid aortic valve

## Abstract

Three patients presented with different symptoms to the emergency department. Further imaging of their hearts displayed an abnormal variant of their aortic valve called a quadricuspid aortic valve (QAV). There are seven types of QAVs, from type A to G, with varying presenting symptoms. The most common complication is aortic regurgitation. The management of QAV is based on these presenting symptoms and complications. Surgical valve repair or replacement is indicated when a QAV becomes symptomatic or a QAV results in ventricular remodeling, which can lead to ventricular dysfunction. Successful surgical repair of QAVs has been shown with both tricuspidization and bicuspidization methods.

## Introduction

The abnormal variant of the aortic valve, also known as quadricuspid aortic valve (QAV), is a rare congenital condition with an incidence of only 0.01 percent, and the first-ever case was reported in 1862 [1]. There are seven different variants of QAV that have been reported so far. Even though aortic regurgitation is the most common complication, it also has an association with other congenital anomalies such as non-obstructive cardiomyopathy, pulmonary valve stenosis, ventricular septal defect, and fibromuscular sub-aortic stenosis. We present a case series of 41-year-old, 29-year-old, and 50-year-old patients who were hospitalized with different complaints but eventually diagnosed with quadricuspid aortic valves.

## Case presentation

**Figure 1:**
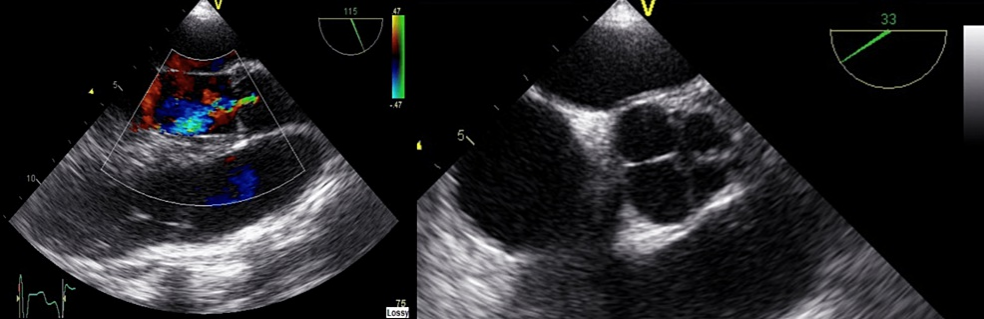
Aortic valve regurgitation on transesophageal echocardiogram (TEE) long axis aortic valve view (left) and quadricuspid aortic valve on TEE short axis aortic valve view (right).

**Figure 2:**
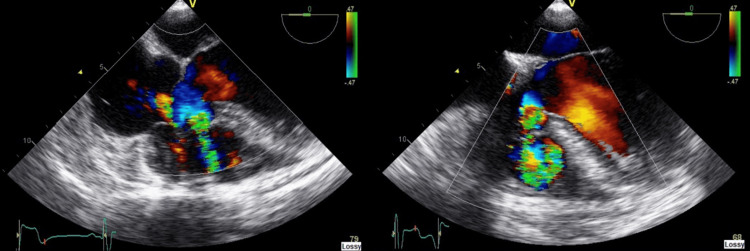
Ventricular septal defect (right and left).

Case 1:

A 41-year-old male with a past medical history of hyperlipidemia, obstructive sleep apnea, hypertension, third-degree atrioventricular block, and right bundle branch block presented to the hospital with gradual onset of shortness of breath, which was aggravated by exercise and alleviated by rest. A diastolic murmur was auscultated on physical examination. Transesophageal echocardiogram (TEE) showed severe aortic valve regurgitation and a quadricuspid aortic valve (Figure 1). TEE also showed ventricular septal defect (VSD) (Figure 2). The patient underwent aortic valve replacement with VSD closure, which improved his dyspnea.

**Figure 3:**
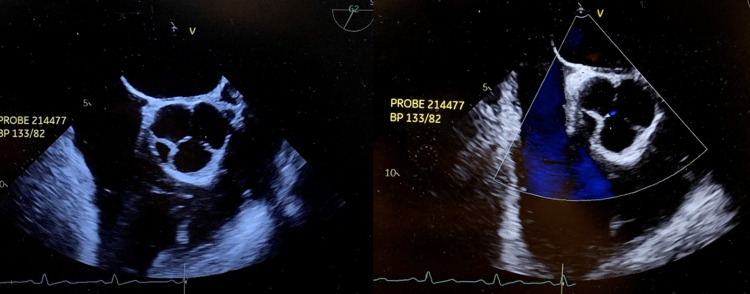
Quadriscuspid aortic valve on transesophageal echocardiogram (TEE) short axis view (right and left).

Case 2:

A 29-year-old male with a past medical history of hepatitis C, splenectomy, and IV drug abuse presented with a right lower extremity infection to the hospital. The patient denied chest pain, palpitations, dyspnea, orthopnea, fatigue, dizziness, and syncope/pre-syncope symptoms. The patient had elevated troponins during the hospital course, which warranted a cardiac workup. An incidental finding on the echocardiogram showed a quadricuspid aortic valve (Figure 3).

**Figure 4:**
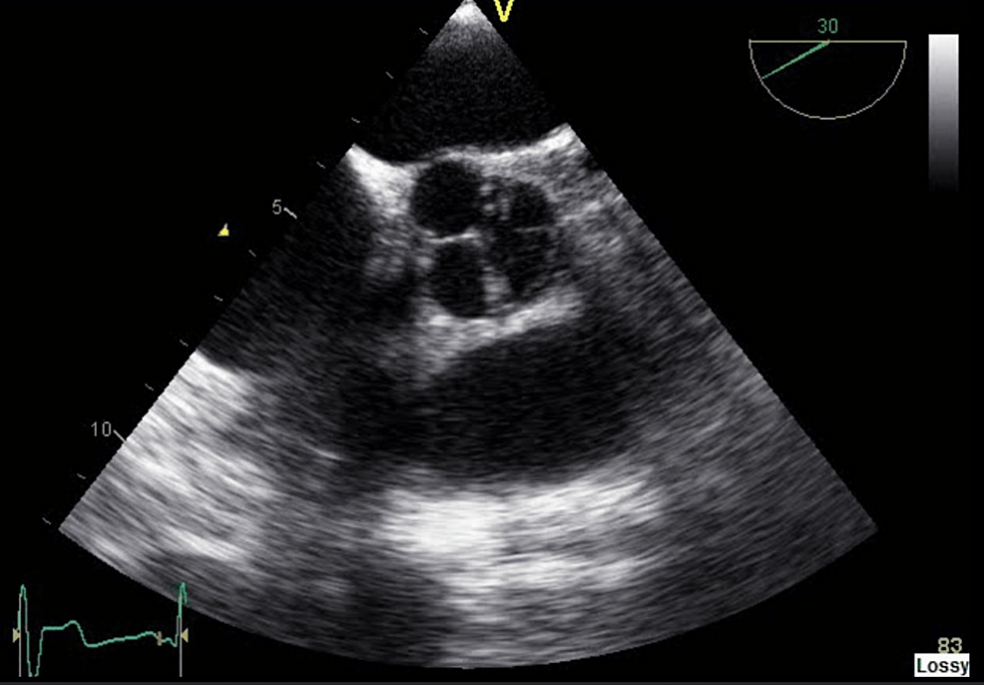
Quadricuspid aortic valve on transesophageal echocardiogram (TEE) short axis view.

Case 3:

A 50-year-old male with no significant past medical history presented to the emergency department with sudden onset of constant palpitations without aggravating or alleviating factors. The patient had one prior similar episode a few months ago, but at that time, he didn’t seek any medical attention. The patient denied chest pain, dyspnea, dizziness, syncope/pre-syncopal symptoms, and recent caffeine and alcohol intake use. The patient was diagnosed with atrial fibrillation, and during cardioversion, he was found to have a quadricuspid aortic valve (Figure 4).

## Discussion

The two tri-leaflet semilunar heart valves include aortic and pulmonary valves developed from endocardial cushions and composed of distinct interstitial cells and stratified extracellular matrix and covered by an outer layer of an endothelial cell. They are formed by different types of progenitor cells that originate inside and outside the heart with organized valve morphology formed through complex signaling [1]. Each leaflet is named based on its relationship with coronary arteries. Unicuspid, bicuspid and quadricuspid are the three different variants of the aortic valve, with the most common variant being bicuspid, followed by unicuspid, and quadricuspid being the rarest [2,3]. The formation of the semilunar valve begins with two different mesenchymal ridges descending to form an aorticopulmonary septum at the junction of conus and truncus, and three mesenchymal ridges’ swelling growing to create a triangular valve in the ninth week of gestation [3]. The embryology of QAV is still unclear, but it is believed that the abnormal division of one of the three mesenchymal ridges and abnormal septation that usually gives rise to an extra aortic valve leads to the formation of QAV [3-5].

**Figure 5:**
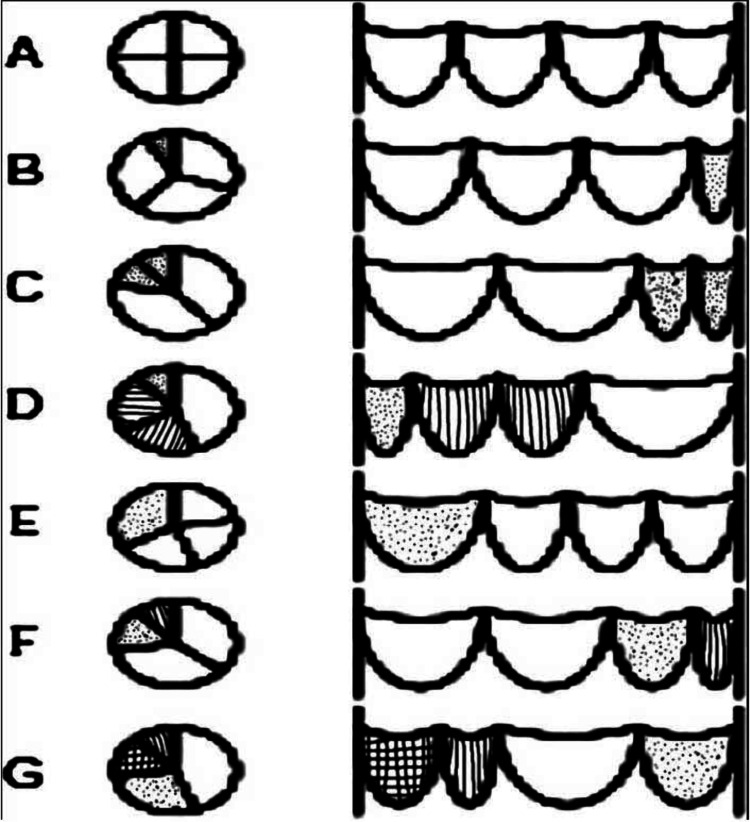
Hurwitz and Roberts' classification of quadricuspid aortic valve. Image source: Oladiran et al., 2019 [2] (Open access)

QAV is congenital or acquired by diseases such as infective endocarditis and rheumatic fever. The absence of corpus arantii (nodules of semilunar cusps) can help differentiate from true genetic versus an acquired QAV. Even though QAV has four points, Hurwitz and Roberts further characterized them into seven different subtypes, A through G, which include the other variant of issues such as type A with four equal points, type B with three similar cusps along with one more minor point, type C with two equally large and small issues, type D with one large and small with two intermediate points, type E with one large and three equal cusps, type F with equally large and two unequal cusps, and lastly type G with four unequal thresholds (Figure 5) [2,3].

QAV has various presentations, from asymptomatic to severe aortic insufficiency. Usually, the patient remains asymptomatic until the fourth or fifth decade of life. The symptoms such as chest pain, dyspnea, congestive heart failure, fatigue, ischemic stroke, and sudden cardiac death have been reported in severe cases. The diagnosis is made via echocardiography (transthoracic/transesophageal). Two-dimensional (2-D) transthoracic echocardiogram provides information about aortic valve morphology, such as cusps, the vegetation of valves, and thickness, along with its function like regurgitation or stenosis. Currently, a transesophageal echocardiogram is favored over a transthoracic echocardiogram, which provides the valve’s morphology and shows coronary abnormalities. CT is also a diagnostic tool but is not as accurate in delivering leaflets and aortic regurgitation. Lastly, Cardiac MRI can also be used as a diagnostic tool and can show the volume of aortic regurgitation and calcification [6].

Cardiac valve disorder is associated with many health problems and death worldwide, despite surgical and procedural advancements. The management of QAV is based primarily on presenting symptoms and associated complications. Indications for surgical repair include symptomatic QAV or when QAV results in ventricular dysfunction or remodeling. Eventually, the aortic valve replacement is the treatment of choice. Successful valve repair has been reported with tricuspidization and bicuspidization methods, especially QAV type B [4,7,8].

## Conclusions

QAV is a congenital heart variant that can lead to severe complications. It can occur as an isolated incidence or in conjunction with other congenital anomalies. Once diagnosed, asymptomatic patients only require regular follow-ups compared to patients with severe symptomatic aortic insufficiency, who benefit from early intervention with aortic valve replacement at an appropriate time before complications such as left ventricular decompensation develop.

## References

[1] MacGrogan D, Luxán G, Driessen-Mol A, Bouten C, Baaijens F, de la Pompa JL (2014). How to make a heart valve: from embryonic development to bioengineering of living valve substitutes. Cold Spring Harb Perspect Med.

[2] Oladiran O, Nwosu I, Dhital R, Ezioma G (2019). Quadricuspid aortic valve: report of two cases and brief review. Case Rep Cardiol.

[3] Vasudev R, Shah P, Bikkina M, Shamoon F (2016). Quadricuspid aortic valve: a rare congenital cause of aortic insufficiency. J Clin Imaging Sci.

[4] Yuan SM (2016). Quadricuspid aortic valve: a comprehensive review. Braz J Cardiovasc Surg.

[5] Douglas A, Patel A, Batsides G, Safi L (2020). Quadricuspid aortic valve: a rare cause of aortic regurgitation. CASE (Phila).

[6] Tao G, Kotick JD, Lincoln J (2012). Heart valve development, maintenance, and disease: the role of endothelial cells. Curr Top Dev Biol.

[7] Kariyanna PT, Francois J, Jayarangaiah A, Chowdhury YS, Grodman R, Salifu MO, McFarlane IM (2020). Quadricuspid aortic valve: a case report and review. Am J Med Case Rep.

[8] Veronese ET, Brandão CM, Steffen SP, Pomerantzeff P, Jatene FB (2019). Quadricuspid aortic valve: three cases report and literature review. Braz J Cardiovasc Surg.

